# Turmeric extract gel and honey in post-cesarean section wound healing: A preliminary study

**DOI:** 10.12688/f1000research.134011.1

**Published:** 2023-09-01

**Authors:** Andi Nilawati Usman, Sartini Sartini, Risfah Yulianti, Melani Kamsurya, Agriyaningsih Oktaviana, Zafitri Nulandari, Dinah Inrawati Agustin, Fendi Fendi

**Affiliations:** 1 Midwifery, graduate school, Universitas Hasanuddin, Makassar, South Sulawesi, 90245, Indonesia; 2 Faculty of Pharmacy, Hasanuddin University, Indonesia, Universitas Hasanuddin, Makassar, South Sulawesi, 90245, Indonesia; 3 Research Institute and Community Service, Wuna Agricultural Sciences Univerisity, Wuna, Indonesia

**Keywords:** Honey Gel, Trigona, Turmeric Extract Gel, and Wound Healing Process

## Abstract

**Background**: Delivery by cesarean section (SC) increases the risk of a surgical site infection (SSI). Therapy from health services and complementary therapy reduce the risk of infection and accelerate the wound-healing process. This study compared wound healing after SC with a turmeric extract gel and original Trigona honey.

**Methods**: Female white rats ( *Rattus novergicus*) with pre- and post-testing and a control group were included in this experiment, which was conducted in June-July 2022. The test animals were 56 female white rats, 2-4 months old, weighing 150–350 g. The treatment group was divided into three subgroups with application of 50% and 75% turmeric extract gel and Trigona honey. The turmeric was given twice daily, and the honey was divided into two applications of twice a day and once a day. Wounds were assessed using the Reeda Scale.

**Results**: The fastest wound healing occurred in the group given Trigona honey twice daily. Redness, ecchymosis, and edema disappeared in this group on day 9 (score 0), and granulation tissue formed on day 9. The group that was administered 50% and 75% turmeric gel extract and Trigona honey once a day healed by days 12 and 15, respectively; all three of these interventions were better than the control group.

**Conclusions**: Administering Trigona honey twice daily was more effective for accelerating wound healing than the 50% or 75% turmeric extract gel. Original Trigona honey has the potential to be a post-SC wound healing agent.

## Introduction

Delivery by Cesarean section (SC) increases the risk of surgical site infection (SSI), which can prolong the hospital stay and increase anxiety. Sepsis can follow if the infection is not resolved. The choice of surgical technique and antibiotic reduces this risk; however, particular risk factors, such as anemia, obesity, hypertension, and parity, continue to make SSI extremely prevalent. ^1^^,^^2^

*Staphylococcus aureus* is the most prevalent pathogenic bacteria in patients with SSI, and 9.8% of these bacteria are resistant to methicillin. Other bacteria include *Klebsiella pneumoniae*, *Escherichia coli*, and *Enterococcus faecalis.* ^3^^,^^4^ Antibiotics, such as azithromycin, reduce the risk of SSI, although some studies suggest that the long-term effects of using antibiotics are unclear. ^5^

Complementary therapy based on natural ingredients has received attention because consumers tend to prefer natural ingredients. ^6^^,^^7^ The local wisdom of Indonesian people, especially those in South Sulawesi, a province in the eastern part of Indonesia, and various studies have shown that honey and turmeric have anti-inflammatory activities and reduce the expression of proinflammatory cytokines. Treating wounds with honey significantly increases angiogenesis, re-epithelialization, and the formation of granulation tissue. The phenolic and flavonoid compounds in honey and turmeric play a role in wound healing. ^7^^–^^9^

Turmeric is a yellow rhizome that is often used as a cooking spice. Turmeric contains active compounds, such as curcuminoids and essential oils. Curcuminoids consist of curcumin, desmethoxycumin, and bisdesmethoxycurcumin. ^10^ The mechanism of action of turmeric extract on wounds is to inhibit cyclooxygenase (COX-2) and lipoxygenase, which play roles in the inflammatory stage to accelerate tissue re-epithelialization, cell proliferation, and collagen synthesis. ^11^

The antibacterial activity of honey is exhibited by components such as H _2_O _2_, glucose, and several polyphenols. The phenolic components inhibit bacterial growth through pro-oxidative activity by increasing H _2_O _2_ activity. The acidic pH of honey (3.2–4.5) also functions as an antibacterial due to the presence of gluconate acid from the oxidation of glucose, which creates an acidic environment. The flavonoid and phenolic components are anti-inflammatory and suppress the activity of the proinflammatory cytokines, such as COX-2 and inducible nitric oxide synthase. Proline, arginine, methionine, and glycine contribute to the formation of collagen and fibroblasts during wound healing. ^8^ Honey has been clinically applied successfully to treat elderly wounds that do not heal after homecare services. Scar tissue has also been treated successfully at the preclinical trial stage. Significant changes in angiogenic biomarkers occur, such as hypoxia-inducible factor-1α, vascular endothelial growth factor (VEGF), and VEGF receptor-II. Honey is also effective on diabetic wounds, burns, pressure sores, and venous and arterial ulcers. ^12^^–^^14^

Turmeric gel and honey have been preclinically tested on post-SC wounds and produced satisfactory results. Researchers tested turmeric gel and applied pure honey to general wounds. We continued this research on more specific wounds, such as postoperative SC wounds in experimental animals. The turmeric was formulated in 50% and 75% gel products, whereas the honey used was 100% pure honey from *Apis trigona* bees used once or twice a day. Both were made with local ingredients from South Sulawesi Province, Indonesia.

This study used female Wistar strain *Rattus norvegicus* rats because they have a gene structure and physiology similar to humans. We compared wound healing, such as redness, swelling, ecchymosis, edema, pus, and tissue granulation, between the turmeric extract gel and pure Trigona honey. This study will hopefully become the basis for clinical trials to prevent SSI in post-SC wounds.

## Methods

### Research design and ethics approval

This research was an experimental pre-post-test laboratory study with female rats ( *R. novergicus*) using a control group. This research received approval from the ethical clearance commission of Hasanuddin University Makassar (number 4883/UN4.14.1/TP.01.02/2022).

### Research setting

The first stage of the research was carried out at the Hasanuddin University Biopharmaceutical Laboratory to manufacture the turmeric gel extract and the honey. The husbandry and treatment of the experimental animals were carried out at the Animal Laboratory, Faculty of Veterinary Medicine, Hasanuddin University Makassar. This study was conducted during June–July 2022.

### Research subjects

The testing was carried out on 56 female white rats. The inclusion criteria for the study were female Wistar white rats, 2–4 months of age, weighing 150–350 g in good health (actively moving, fur not dull, not shedding, eyes clear, and nimble). The exclusion criteria were illness and inactivity throughout the study.

Thirty-one rats did not meet the inclusion criteria (28 rats under 150 g, and three were not active and ill), so 25 rats (n=5/group) were used in the study.

### Research procedure

All efforts were undertaken to minimize the suffering of the animals. Wounds were created after shaving the fur on the part to be cut. The rats were anesthetized intraperitoneally with ketamine (80 mg/kg) before wounding to relieve pain and prevent excessive movement. The area to be cut was marked, and a 2 cm-long incision was made to penetrate the peritoneum. Five groups were prepared:

1. Turmeric gel at 50% and 75% concentrations were applied to the wound using sterile cotton every morning and evening
2. Trigona honey gel was applied twice daily (0.13 mg) (morning and evening) or once daily (0.064 mg) in the morning to the respective subgroups
3. Control, without intervention

The wound healing was monitored by observing moisture, redness, edema, pus, and granulation tissue in the wound (Reeda Scale). The rats were euthanized after closing the intrathoracic transection of the great vessels and heart. ^15^

## Data analysis

Data were grouped according to purpose and type, and descriptive statistical methods were used. The characteristics of the basic data are described to see improvements and no statistics were used. The score entered in the table is the average score (mean) which is calculated by adding up all the female Wistar white rat wound scores in each group, then dividing it by the number of group members. The score was calculated using Microsoft Excel.

### Research results

Table 1 shows that the groups given the 50% and 75% turmeric extract gel had a redness score of 0 (redness disappeared) on day 12 post-intervention. The groups that were given Trigona honey twice daily and once daily had scores of 0 (redness disappeared) 3 days earlier than the group that was given turmeric, which was on day 9.

**Table 1.** Redness score of the wounds in the turmeric extract gel and honey groups.

Intervention group		Score redness of the wounds							
		Day							
		0	3	6	9	12	15	18	21
50% Turmeric extract gel	Score	1.0	1.0	1.0	1.0	0.0	-	-	-
75% Turmeric extract gel	Score	1.0	1.0	1.0	1.0	0.0	-	-	-
Trigona twice a day	Score	1.0	1.0	1.0	0.0	-	-	-	-
Trigona once a day	Score	1.0	1.0	1.0	0.0	-	-	-	-
Control	Score	1.0	1.0	1.0	1.0	0.6	0.0	-	-

Source: Descriptive.

Table 2 shows that none of the groups administered the 50% or 75% turmeric extract gel scored 0 (ecchymosis disappeared) on day 15 post-intervention. The group given Trigona honey twice daily had a score of 0 (ecchymosis disappeared) on day 9, whereas the group given Trigona honey once daily had a score of 0 (ecchymosis disappeared) on day 15.

**Table 2.** Ecchymosis scores of the wounds in the turmeric extract gel and honey groups.

Intervention group		Ecchymosis of the wounds							
		Day							
		0	3	6	9	12	15	18	21
50% Turmeric extract gel	Score	3.0	3.0	1.6	1.2	1.0	0.0	0.0	0.0
75% Turmeric extract gel	Score	3.0	3.0	1.4	1.2	1.0	0.0	0.0	0.0
Trigona twice a day	Score	3.0	2.0	1.0	0.0	-	-	-	-
Trigona once a day	Score	3.0	2.0	2.0	1.0	1.0	0.0	-	-
Control	Score	3.0	3.0	1.6	1.4	1.2	1.0	0.0	0.0

Source: Descriptive.

Table 3 shows that the groups administered the 50% or 75% turmeric extract gel had scores of 0 (redness disappeared) on day 12 postintervention. The group given Trigona honey twice daily had a score of 0 (redness disappeared) 3 days earlier than the group given turmeric on day 9 (redness disappeared).

**Table 3.** Edema score of the wounds in the turmeric extract gel and honey gel groups.

Intervention groups		Edema of the wounds							
		Day							
		0	3	6	9	12	15	18	21
50% Turmeric extract gel	Score	1.0	1.0	1.0	1.0	0.0	-	-	-
75% Turmeric extract gel	Score	1.0	1.0	1.0	1.0	0.0	-	-	-
Trigona twice a day	Score	1.0	1.0	1.0	0.0	-	-	-	-
Trigona once a day	Score	1.0	1.0	1.0	1.0	0.0	-	-	-
Control	Score	1.0	1.0	1.0	1.0	0.6	0.0		

Source: Descriptive.

Table 4 shows that the control group displayed pus on day 12 and scored 0 (pus disappeared) on day 15 post-intervention, whereas none of the other groups experienced pus in a wound.

**Table 4.** Pus score of the wounds in turmeric extract gel and honey gel groups.

Intervention groups		Pus of the wounds							
			Day						
		0	3	6	9	12	15	18	21
50% Turmeric extract gel	Score	-	-	-	-	-	-	-	-
75% Turmeric extract gel	Score	-	-	-	-	-	-	-	-
Trigona twice a day	Score	-	-	-	-	-	-	-	-
Trigona once a day	Score	-	-	-	-	-	-	-	-
Control	Score	-	-	-	-	1.0	0.0	-	-

Source: Descriptive.

Table 5 shows that granulation occurred the quickest (score 2) in the group given Trigona honey twice daily, whereas the group given 50% or 70% turmeric gel extract experienced granulation (score 2.0) on day 15; the longest time to granulation was the control group on day 18.

**Table 5.** Granulation tissue score in the wound of the turmeric extract gel and honey groups.

Intervention groups		Granulation tissue in wounds							
		Day							
		0	3	6	9	12	15	18	21
50% Turmeric extract gel	Score	0.0	0.6	1.0	1.2	1.8	2.0	-	-
75% Turmeric extract gel	Score	0.0	0.6	1.0	1.4	1.8	2.0	-	-
Trigona twice a day	Score	0.0	1.2	1.8	2.0	-	-	-	-
Trigona once a day	Score	0.0	0.0	1.0	1.2	1.8	2.0	-	-
Control	Score	0.0	0.4	0.8	1.0	1.4	1.8	2.0	-

Source: Descriptive.

## Discussion

Drugs have been used to speed up the wound-healing process. Wound healing is a natural repair process for tissue injury involving inflammatory mediators, blood cells, the extracellular matrix, and parenchymal cells. ^16^ The drugs used in traditional medicines are derived from plants and animals. ^17^ Some of the plants used to treat wounds in mice include turmeric and honey. ^18^

The present study showed that administering Trigona honey twice daily accelerated wound healing based on redness, moisture, edema, granulation tissue, and scabs in the wound compared with the 50% and 75% turmeric gel extract groups ( Tables 2– 5). Trigona honey administered twice daily did not result in wound edema ( Table 4). Honeys have different chemical compositions, biological properties, and effects depending on the environmental, geographical, and nutritional factors of the plant pollen collected. ^17^ Trigona honey increases angiogenic activity, which is very important during wound healing and accelerates the formation of granulation tissue and re-epithelialization of the skin. Honey has antioxidant, antibacterial, and anti-inflammatory properties. ^18^ The flavonoid content in honey increases angiogenesis; thus, fibrosis and collagen formation increase. Flavonoids also have antibacterial, antioxidant, and anti-inflammatory properties. ^19^

The results of this study indicate that administering Trigona honey twice daily led to a faster healing process than once daily; this agreed with research conducted by Takzaree *et al.*, who reported that applying honey to wounds twice daily increases healing, shortens the inflammatory process, and increases the granulation rate. ^20^

Turmeric extracts in 50% and 75% gels can be used as an alternative treatment because they accelerate wound healing, although not as quickly as Trigona honey ( Tables 2– 5). This is due to the curcumin compounds with antimicrobial, antioxidant, and anti-inflammatory properties that accelerate re-epithelialization, proliferation, and collagen synthesis. ^21^^–^^23^ Turmeric rhizome extracts have antibacterial activity. Turmeric leaf oil at various concentrations inhibits the mycelial growth of *Aspergillus flavus* and *Aspergillus parasiticus.* Turmeric leaf oil is effective against the antibiotic-resistant bacterium *Escherichia coli*, and the oil from *Curcuma longa* leaves has antioxidant properties. ^23^^–^^25^ The turmeric rhizome is prepared as a gel for topical application. A gel is a semisolid system that provides a cool and soothing feeling to the skin with a high water content so it increases hydration in the stratum corneum and dries easily to form a film layer. ^11^ The results of this study indicate that the turmeric extract gel and Trigona honey can be used as alternative treatments for post-CS wounds. However, further research is needed on humans to determine the appropriate dose.

## Author contributions

ANU, SAR, RFY, MNK, AGR, amf ZAN contributed to the literature review, data extraction from various databases, conceptualisation, development of the economic models on Microsoft Excel Software, formal analysis, findings interpretation, and manuscript writing. All authors approved the final version of the paper.

## Data Availability

Figshare: MASTER TABLE OF WOUND HEALING OF SECTIO CAESAREA IN RATS.xlsx, https://doi.org/10.6084/m9.figshare.22756712 This project contains the following underlying data:
- MASTER TABLE OF WOUND HEALING OF SECTIO CAESAREA IN RATS.xlsx - TURMERIC EXTRACT GEL IN POST SECTIO SECAREA WOUND HEALING.xlsx Data are available under the terms of the Creative Commons Zero “No rights reserved” data waiver (CC0 1.0 Public domain dedication).
