# Supplementary material for: Turmeric extract gel and honey in post-cesarean section wound healing: A preliminary study
Source: F1000Res. 2024 Feb 20;12:1095. Originally published 2023 Sep 1. [Version 2] doi: 10.12688/f1000research.134011.2 (PMC11137484; doi:10.12688/f1000research.134011.2)
Supplement: Peer review history

# Reviewer report: Reviewer response for version 2

**DOI:** 10.5256/f1000research.162529.r275078

**Published:** 2024-05-29

**Authors:** Evelyn Asante-Kwatia

**Affiliations:** 1 Department of Pharmacognosy, Faculty of Pharmacy and Pharmaceutical Sciences, College of Health Sciences, Kwame Nkrumah University of Science and Technology, Kumasi, Ashanti Region, Ghana

All methods used are scientifically valid; and analytical tools for results analysis as technically accurate. The authors have clearly described all protocols in detail to make them reproducible. Ethical approvals were sought for the study and provided. The manuscript has been well-written.

 It is important that authors highlight the novelty of the work in the conclusion.

Is the work clearly and accurately presented and does it cite the current literature?

Yes

If applicable, is the statistical analysis and its interpretation appropriate?

Yes

Are all the source data underlying the results available to ensure full reproducibility?

Yes

Is the study design appropriate and is the work technically sound?

Yes

Are the conclusions drawn adequately supported by the results?

Yes

Are sufficient details of methods and analysis provided to allow replication by others?

Yes

Reviewer Expertise:

ethnobotany, pharmacognosy, phytochemistry

I confirm that I have read this submission and believe that I have an appropriate level of expertise to confirm that it is of an acceptable scientific standard.

# Reviewer report: Reviewer response for version 2

**DOI:** 10.5256/f1000research.162529.r252100

**Published:** 2024-03-19

**Authors:** Arfa Nasrine

**Affiliations:** 1 Department of Pharmaceutics, College of Pharmaceutical Sciences Dayananda Sagar University, Bengaluru, Karnataka, India

Can be accepted

Reviewer Expertise:

Pharmaceutical novel formulation development

I confirm that I have read this submission and believe that I have an appropriate level of expertise to confirm that it is of an acceptable scientific standard.

## References

[1] : Turmeric extract gel and honey in post-cesarean section wound healing: A preliminary study. *F1000Research* .2024;12: 10.12688/f1000research.134011.2 10.12688/f1000research.134011.2 PMC1113748438817411

# Reviewer report: Reviewer response for version 1

**DOI:** 10.5256/f1000research.147035.r232732

**Published:** 2024-01-31

**Authors:** Arfa Nasrine

**Affiliations:** 1 Department of Pharmaceutics, College of Pharmaceutical Sciences Dayananda Sagar University, Bengaluru, Karnataka, India

Title: Review of Manuscript - "Turmeric extract gel and honey in post-cesarean section **wound healing: A preliminary study"

 Overall Impression:

 The manuscript presents an interesting study on the use of turmeric extract gel and *Trigona *honey for wound healing in post-Cesarean section (SC) wounds in rats. The study design, methodology, and results are well-documented, providing valuable insights into the potential benefits of these natural products in wound healing. However, there are a few points that need clarification and improvement.

 1. Title and Keywords:

 The keywords adequately capture the main topics of the manuscript, but it might be helpful to include terms like "Cesarean section" and " *Rattus norvegicus*" for enhanced discoverability.

 2. Introduction:

 a. The introduction effectively establishes the background and rationale for the study. However, it would be beneficial to provide more recent statistics or studies related to surgical site infections (SSI) and the use of alternative therapies.

 b. The section on *Staphylococcus aureus* and antibiotic resistance is well-presented. However, it would be useful to include references to support the mentioned prevalence rates and resistance percentages.

 3. Literature Review:

 a. The literature review provides a comprehensive overview of the anti-inflammatory properties of honey and turmeric. However, including recent references and studies, especially those related to wound healing, would strengthen this section.

 b. Clarify whether the studies mentioned are specific to the *Trigona* honey from Apis trigona bees, as this distinction is crucial.

 4. Methods:

 a. The research design is clearly described, but it would be helpful to provide a brief rationale for choosing the Wistar strain *Rattus norvegicus* rats, considering the similarity of their gene structure and physiology to humans.

 b. Include information on the randomization process for assigning rats to different intervention groups.

 c. Specify the blinding procedures, if any, during the wound healing assessments.

 5. Results:

 a. The presentation of results in tables is clear and organized. However, consider providing standard deviations or confidence intervals for the reported mean scores to assess the variability within each group.

 b. The discussion of the results should reference the relevant tables for better clarity.

 6. Discussion:

 a. The discussion provides a good interpretation of the results. However, the comparison of the effectiveness between turmeric extract gel and *Trigona* honey could be more nuanced. Consider discussing potential synergistic effects of combining these treatments.

 b. The limitations of the study should be explicitly mentioned, such as the use of animal models and the need for further human trials.

 7. Data Availability:

 a. Ensure that the data availability statement is accurate, and the provided DOI links lead to the correct datasets.

 8. Language and Style:

 a. The manuscript is generally well-written, but some sentences could be clarified for better readability. Consider revising for conciseness and clarity.

 9. Conclusion:

 The manuscript presents a promising study on the potential benefits of turmeric extract gel and *Trigona* honey in wound healing. Addressing the mentioned points would enhance the manuscript's overall quality and impact.

Reviewer Expertise:

Pharmaceutical novel formulation development

I confirm that I have read this submission and believe that I have an appropriate level of expertise to confirm that it is of an acceptable scientific standard, however I have significant reservations, as outlined above.

# Reviewer report: Reviewer response for version 1

**DOI:** 10.5256/f1000research.147035.r224741

**Published:** 2023-11-27

**Authors:** Maulana Yusuf Alkandahri

**Affiliations:** 1 Universitas Buana Perjuangan Karawang, Karawang Regency, West Java, Indonesia

1. Please complete all your bio-assay with completed validation methods (Please read https://doi.org/10.1016/j.jpba.2022.114735, it maybe can help you to revise your manuscript (at least please calculate Z factor of your methods). Without validation data, the reported results maybe not be replicated by other researchers).
2. Please delete all significance tests, and replace it with data of (mean ± Confidence interval) (see Gandevia, S., Cumming, G., Amrhein, F., Butler, A. 2021. Replication: Do not trust your p-value, be it small or large. J. Physiol. 599(11): 2989-2990.
3. The study groups, the pattern of using the extract, and administration intervals should be given in a separate section under the title of "treatment groups". Also, the protocol and study design should be added as a graphic schematic design to help the author's inference.
4. This manuscript needs editing in English language and grammar by a native English speaker. Also, a major spacing problem were noticed all over the text. Please consider thorough editing on that aspect.
5. Overall scientific term also needs to be edited. They are very informally written, which is not acceptable for publishing in a scientific journal in current format.
6. 80% of the references cited should be between 2018-2023.
7. The revised and corrected manuscript should be subjected to plagiarism checker (<10%).

Is the work clearly and accurately presented and does it cite the current literature?

Yes

If applicable, is the statistical analysis and its interpretation appropriate?

I cannot comment. A qualified statistician is required.

Are all the source data underlying the results available to ensure full reproducibility?

Partly

Is the study design appropriate and is the work technically sound?

Yes

Are the conclusions drawn adequately supported by the results?

Partly

Are sufficient details of methods and analysis provided to allow replication by others?

Partly

Reviewer Expertise:

Molecular Pharmacology

I confirm that I have read this submission and believe that I have an appropriate level of expertise to confirm that it is of an acceptable scientific standard, however I have significant reservations, as outlined above.

## References

[1] : The importance of method validation in herbal drug research. *J Pharm Biomed Anal* .2022;214: 10.1016/j.jpba.2022.114735 114735 10.1016/j.jpba.2022.114735 35344789

[2] : Replication: Do not trust your p‐value, be it small or large. *The Journal of Physiology* .2021;599(11) : 10.1113/JP281614 2989-2990 10.1113/JP281614 33963767
